# Colonisation with ESBL-producing and carbapenemase-producing *Enterobacteriaceae,* vancomycin-resistant enterococci, and meticillin-resistant *Staphylococcus aureus* in a long-term care facility over one year

**DOI:** 10.1186/s12879-015-0880-5

**Published:** 2015-04-01

**Authors:** Catherine Ludden, Martin Cormican, Akke Vellinga, James R Johnson, Bernie Austin, Dearbháile Morris

**Affiliations:** Discipline of Bacteriology, School of Medicine, National University of Ireland Galway, Galway, Ireland; Centre for Health from Environment, Ryan Institute, National University of Ireland Galway, Galway, Ireland; VA Medical Center, Minneapolis, MN USA; St. Brendan’s Home, Community Nursing Unit, Galway, Ireland

**Keywords:** Extended-spectrum beta-lactamase (ESBL), Carbapenemase-producing *Enterobacteriaceae* (CPE), Vancomycin-resistant enterococci (VRE), Meticillin-resistant *S. aureus* (MRSA), Long-term care facilities

## Abstract

**Background:**

This study examined colonisation with and characteristics of antimicrobial-resistant organisms among residents of a long-term care facility (LTCF) over one year, including strain persistence and molecular diversity among isolates of extended-spectrum beta-lactamase (ESBL)-producing *Enterobacteriaceae*.

**Methods:**

Sixty-four residents of a LTCF were recruited (51 at baseline, 13 during the year). Data on dependency levels, hospitalisations, and antimicrobial prescribing were collected. Nasal and rectal swabs and catheter urine specimens were examined quarterly, using chromogenic agars, for ESBL-producing *Enterobacteriaceae*, carbapenemase-producing *Enterobacteriaceae* (CPE), vancomycin-resistant enterococci (VRE), and meticillin-resistant *S. aureus* (MRSA). All ESBL-producing *E. coli* (ESBL-EC) were characterised by pulsed-field gel electrophoresis (PFGE) and PCR to assess for sequence type (ST) ST131, its resistance-associated *H*30 and *H*30-Rx subclones, and *bla*_CTX-M,_*bla*_TEM,_*bla*_SHV,_ and *bla*_OXA-1_.

**Results:**

The overall number of residents colonised, by organism, was as follows: ESBL-EC, 35 (55%); MRSA, 17 (27%); ESBL-producing *K. pneumoniae* (ESBL-KP), 5 (8%); VRE, 2 (3%) and CPE, 0 (0%). All 98 ESBL-EC isolates were *H*30-Rx ST131, with *bla*_CTX-M-group 1._ By PFGE, a group of 91 ESBL-EC (from 33 participants) had ≥ 85% similar profiles and resembled UK epidemic strain A/ international pulsotype PFGE812. Sequential ESBL-EC from individual residents were closely related. Six ESBL-KP isolates, from five participants, had *bla*_CTX-M-group 1_ and by PFGE were closely related. Colonisation with ESBL and MRSA was associated with location within the LTCF and previous exposure to antimicrobials.

**Conclusions:**

Among LTCF residents, colonisation with ESBL-EC and MRSA was common. All ESBL-EC were *H*30-Rx ST131, consistent with clonal dissemination.

## Background

Antimicrobial resistance has been identified as a key public health challenge [1]. Amongst the major acquired antimicrobial-resistant organisms (AROs) are extended-spectrum beta-lactamase (ESBL)-producing *Enterobacteriaceae*, carbapenemase-producing *Enterobacteriaceae* (CPE), vancomycin-resistant enterococci (VRE), and meticillin-resistant *Staphylococcus aureus* (MRSA).

ESBL-producing *E. coli* (ESBL-EC) have become increasingly common throughout the world. In Ireland, ESBL-EC represented 10% of all *E. coli* bloodstream infections in 2013, compared with 3% in 2006 [2]. Spread of CTX-M beta-lactamases is linked with specific epidemic clonal groups such as *E. coli* sequence type ST131 (typically of serotype O25b:H4). Although ST131 has been identified from as early as 1967, ST131 isolates containing ESBLs, predominantly CTX-M-15, emerged mainly in the 2000s [3,4]. There is significant genomic diversity within ST131, with over 170 distinct PFGE patterns/pulsotypes (>94% similar *Xba*I PFGE profiles) recognised, some of which are associated with particular sources and antimicrobial resistance patterns [3].

The *H*30 ST131 subclone, so named for its carriage of allele 30 of *fimH* (type 1 fimbrial adhesin gene), reportedly expanded and disseminated rapidly after 2000 to become the most common subclone among clinical *E. coli* isolates globally [5,6]. *H*30 is closely associated with fluoroquinolone resistance [5,6]. ESBL-EC, most of which carry *bla*_CTX-M-15_, belong mainly to a subset within *H*30 known as *H*30-Rx, which is strongly associated with sepsis [5,7].

Analogous to ST131 among ESBL-EC, several widely disseminated clonal groups of ESBL-producing *K. pneumoniae* (ESBL-KP) have also been identified, including *K. pneumoniae* ST11, ST15, ST16, ST23, and ST48 [8-12]. Recently, emerging carbapenem resistance has been reported in both *E. coli* and *K. pneumoniae,* further limiting options for antimicrobial therapy for these organisms [13,14].

Since the first *Klebsiella pneumoniae* carbapenemase (KPC)-producers were reported in Ireland in 2009, and the first KPC-producing *E. coli* in 2011, the numbers of CPE detected are increasing [15,16]. Likewise, VRE has caused an increasing proportion of all enterococcus bloodstream infections in Ireland in recent years [17]. By contrast with the above antimicrobial-resistant organisms, MRSA as a proportion of all *S. aureus* bloodstream infection has declined markedly, although strains isolated remain predominantly the hospital-associated strains that have been prevalent for decades [17,18].

There is increasing evidence that long-term care facilities (LTCFs) are important reservoirs for AROs, including reports of widespread colonisation and outbreaks [19-23]. This phenomenon is likely to increase in importance, since the proportion of the European Union population aged ≥ 65 years is now 16% and will increase to 29.3% (152.6 million) by 2060 [24]. This is likely to further increase the population residing in LTCFs, currently at 19,800 in Ireland (2012) and approximately 3.7 million in the EU (2010) [24,25].

Risk factors for acquisition of AROs such as old age, urinary catheterisation, antimicrobial consumption, and hospitalisation are common among residents of LTCFs [26-30]. Although there are a number of studies of prevalence of AROs in LTCFs, there are few longitudinal studies regarding the stability of colonisation over time, especially in relation to molecular characteristics of colonising organisms [31]. The aims of this study were: (i) to determine the baseline prevalence of colonisation; (ii) to monitor residents’ colonisation status at quarterly intervals over one year; (iii) to identify risk factors for colonisation; (iv) to characterise the antimicrobial susceptibility of the AROs; and (v) to define the molecular characteristics of all ESBL-producing *Enterobacteriaceae* isolates.

## Methods

### Setting

The study was based in a newly built 100-bed LTCF that had 88 residents at the start of the study. There are four discrete care areas, with residents grouped by dependency level. Based on the Barthel Index, residents in care areas 1 and 2 are generally highly dependent for assistance for the 10 activities for daily living care (daily care), whereas those in care areas 3 and 4 require only occasional assistance with daily living (intermittent care). To simplify the models for statistical analysis, the four care areas were collapsed into two groups. Each of the four care areas has 21 single and two double en-suite rooms, a dining area, and a day room. There are two lifts and two sets of stairs.

### Ethical approval

Ethical approval was granted by the Galway University Hospital Ethics Committee. Written consent was obtained at the outset and participants could withdraw from the study at any time.

### Design and data collection

The study period was July 2012-August 2013. For each participating resident, data collected included gender, age on admission, date of admission, from where the resident was admitted, presence of an indwelling urinary catheter, systemic antimicrobial treatment, and hospitalisation in the previous 12 months, and previous ESBL, CPE, VRE, and MRSA result recorded before study commencement. At each quarterly interval additional data were recorded, including recent antimicrobial treatment in the LTCF, number of residents in the same room, hospitalisation, presence of a wound/ulcer, Barthel Index score (dependency Index), and location within the LTCF. However, data regarding antimicrobial treatment received during intercurrent hospitalisation were not accessible. Antimicrobials were classified as: (1) narrow spectrum beta-lactams, (2) broad spectrum beta-lactams, (3) quinolone/fluoroquinolones, (4) nitroimidazole, (5) nitrofurantoin, and (6) others. An antimicrobial day was defined as any day on which a resident received an antimicrobial.

### Clinical sample collection

At quarterly intervals nasal and rectal swabs were collected from each participating resident, and urine samples were obtained from catheterised participants.

### Laboratory detection

Rectal swabs were cultured on chromogenic agar for detection of ESBL-producing *Enterobacteriaceae* and vancomycin resistant enterococcci (ID™ ESBL agar and chrom ID™ VRE agar (bioMérieux, Marcy l’Etoile, France)). The Centers for Disease Control and Prevention (CDC) method was applied to detect CPE [32,33]. Nasal swabs were cultured on chrom ID™ MRSA agar (bioMérieux, Marcy l’Etoile, France). In each case suspect colonies were subcultured for identification by standard methods and susceptibility testing was performed in accordance with EUCAST disk diffusion methods [34].

### Molecular analysis

All ESBL-EC and ESBL-KP isolates were tested by PCR for *bla*_CTX-M,_*bla*_TEM,_*bla*_SHV, and_*bla*_OXA-1_ as described previously [35,36]. *E. coli* were tested for ST131-specific single-nucleotide polymorphisms (SNPs) in *pabB* by PCR and, for isolates testing positive, additional SNP-based PCR reactions were performed to identify the *H*30 and *H*30-Rx subclones [5,37,38]. *Xba*I pulsed-field gel electrophoresis (PFGE) analysis was done according to the Pulse-Net protocol [39]. PFGE profiles were analysed using the Dice coefficient with clustering by the unweighted pair group method with arithmetic averaging (UPGMA). For reference, *E. coli* ST131 isolates representing UK Strains A, C, and D, and international pulsotypes PFGE788, PFGE797, PFGE800, PFGE806, PFGE812, PFGE837, PFGE842, PFGE903, PFGE905, PFGE906, PFGE945, PFGE968, PFGE987, and PFGE1140 were included in the *E. coli* PFGE analysis [3,40]. Similarly, representative *K. pneumoniae* isolates for STs ST14, ST15, ST16, ST23, ST35, ST37, ST45, ST48, ST101, ST147, ST161, ST258, ST280, ST307, ST392, ST429, and ST1236 were included in the *K. pneumoniae* PFGE analysis.

### Environmental sampling

In August 2013, after commencement of the longitudinal study, environmental sampling was performed to detect ESBL-EC, ESBL-KP, VRE, and MRSA. Samples were taken from five en-suite bedrooms and two shared toilet facilities. The criteria for the selection of the five bedrooms was based on the selection of bedrooms of residents who tested positive for carriage of ESBL-EC, VRE and MRSA (n = 1), ESBL-EC and ESBL-KP (n = 1), ESBL-EC only (n = 1), ESBL-KP only (n = 1), and one occupant who did not have detectable colonisation with any of the target organisms (n = 1). The five bedrooms selected were based in two care areas and one common bathroom was selected from each of these two care areas. Eleven sites in each bedroom and seven sites in each shared toilet facility were tested. Selected sites included door handles entering and exiting areas, floor surface at base of toilet, handles beside showers, toilet flushers, toilet seats, tap handles, railings beside toilets, bed-frames, bed-side lockers, and on-call buttons.

Sites were swabbed using Copan ESwabs (BS ISO 18593:2004) and inoculated into peptone water overnight. Ten microlitres of peptone water was plated onto chromID™ MRSA, chromID™ESBL agar and chromID™VRE (bioMerieux) for detection of MRSA, ESBL, and VRE respectively. Identification of suspect isolates was by standard methods and susceptibility testing was interpreted by EUCAST methods and criteria [34].

### Statistical analysis

For ESBL and MRSA colonisation univariate comparisons were made to identify associations with patient characteristics.

Generalised Estimating Equations (GEE) models with an exchangeable correlation structure were used to investigate the longitudinal effects taking dependency between repeated measurements in the same individual into account (repeated individual measures at 0, 3, 6, 9 and 12 months). A forward selection procedure was used to estimate the relation between ESBL/MRSA colonization and specific or general antimicrobial use. Potential confounding effects included in the models were previous colonisation, care area, hospitalisation, age, gender and other patient characteristics. Age and gender were kept in the final model, however other variables were only included if found to be a confounder. Interaction terms were checked but omitted if not significant. P-values <0.05 were considered statistically significant. Results are presented as adjusted odds ratios (OR) (adjusted for potential confounders) with corresponding 95% confidence intervals (CI). Analyses were done using SPSS for Windows version 20 and STATA/IC version 13.0.

## Results

### Participating residents

Sixty-four LTCF residents agreed to participate in this study, including 51 (58% of 88 initial residents) at baseline and 13 (45% of 29 subsequently admitted residents) later in the year. The 51 baseline enrolees had a 76-month median prior length of stay in the LTCF. Thirty-four of these participants remained in the study for the entire study year whereas 17 withdrew early (15 due to ill health or death and 2 refused repeated sampling); however, all but 3 of the 17 had at least two rectal and nasal samples taken prior to withdrawal. All 13 newly admitted residents who were enrolled partway through the study year were followed until the end of the study. Of these participants, 11 entered the LTCF from hospital, one from home, and one from another LTCF. Key participant characteristics are summarised in Table 1. During the study period, 10 baseline participants and one new participant were hospitalised (for from 2 to 40 hospital days each). Additionally, 28 baseline and 5 new participants received antimicrobials while in the LTCF (for from 3 to 203 antimicrobial days each).

**Table 1:** **Demographics and clinical details of the 64 long-term care facility participants**

**Factor**	**Description**	**N**	**% of 64**
**Gender**	Male	34	53
	Female	30	47
**Admission group**	Hospital	6	9
	Long-term care facility	19	30
	Home	39	61
**Diabetic**	Yes	16	25
**Care area**	1&2	30	47
	3&4	34	53
**Urinary catheter**	Yes	9	14
**Wound/ulcer**	Yes	8	13
**Antimicrobials** ^**a**^	In year previous	31	48
**Antimicrobials** ^**b**^	During study	33	52
**Hospitalisation** ^**c**^	In year previous	11	17
**Hospitalisation** ^**d**^	During study	37	58
	**Mean (SD)**	**Min-max**	**Median**
**Age**	80 (10.52)	37-98	80
**Initial barthel index**	33 (33.17)	0-100	17.5

^a-d^Details were only accessible for participants in the long-term care facility during the time period, therefore no information on prior hospitalisation and antimicrobial usage was included for new admissions.

### Detection and characterisation of ESBL-producing *E. coli*

As summarised in Table 2, ESBL-EC were detected in 35 (55%) of the 64 participants, including 28 (80%) by the first rectal swab, and seven (20%) by subsequent rectal swabs. For the 24 positive ESBL-EC participants with at least two available follow-up (3 or more total) swabs, 75% remained positive for ≥ 6 months and 42% remained positive for ≥ 1 year. Most (71%) of the ESBL-EC-colonised participants were from (high-dependency) care areas 1 and 2. Approximately half (51%) of baseline participants remained un-colonised throughout, and when new admissions were taken into account, 45% of residents were not colonised.

**Table 2:** **Results for all participants tested from 0 to 12 months for colonisation with the targeted antimicrobial resistant organisms**

	**Proportion colonised (%)**					
**Resistance phenotype**	**July 2012** ^**f**^	**October 2012** ^**f**^	**January 2013** ^**f**^	**May 2013** ^**f**^	**August 2013** ^**f**^	**Any time point**
**ESBL-EC** ^**a**^	20/51 (39%)	21/51 (41%)^7^	14/50 (28%)	21/49 (43%)^7^	19/45 (42%)^7^	35/64 (55%)
**ESBL** ***-*** **KP** ^**b**^	0/51 (0%)	2/51 (4%)	0/50 (0%)	2/49 (4%)	2/45 (4%)	5/64 (8%)
**CPE** ^**c**^	0/51 (0%)	0/51 (0%)	0/50 (0%)	0/49 (0%)	0/45 (0%)	0/64 0%)
**VRE** ^**d**^	0/51 (0%)	0/51 (0%)	1/50 (2%)	1/49 (2%)	1/45 (2%)	2/64 (3%)
**MRSA** ^**e**^	8/51 (16%)	9/51 (18%)	7/50 (14%)	7/49 (14%)	4/45 (9%)	17/64 (27%)
**Any of above**	24/51 (47%)	25/51 (49%)	17/50 (34%)	23/49 (47%)	20/45 (44%)	39/64 (61%)

^a^ESBL-EC; Extended-spectrum beta-lactamase producing-*Escherichia coli.*

^b^ESBL-KP; Extended-spectrum beta-lactamase-producing-*Klebsiella pneumoniae.*

^c^CPE; Carbapenemase-producing *Enterobacteriaceae.*

^d^VRE; Vancomycin-resistant enterococci.

^e^MRSA; Meticillin-resistant *Staphylococcus aureus.*

^f^Number of participants tested at each interval varied, accounting for participants who died, withdrew from the study, or newly joined the study.

^g^One resident had an ESBL-EC positive urine and rectal culture.

The 98 ESBL-EC isolates were recovered from rectal (n = 95) and urine samples (n = 3) and were uniformly resistant to ciprofloxacin, and susceptible to cefoxitin, ertapenem, meropenem, and gentamicin. They all belonged to the *H*30-Rx ST131 subclone and carried *bla*_CTX-M group 1_. Eighty-five (87%) also contained *bla*_OXA-1_, whereas none contained *bla*_TEM_ or *bla*_SHV_ (Table 3). All 85 *bla*_OXA-1-_positive isolates were co-amoxiclav-resistant, whereas all *bla*_OXA-1_ negative isolates were co-amoxiclav-susceptible.

**Table 3:** **Correlation of pulsed-field gel electrophoresis (PFGE) clusters with** ***bla*** **type, sequence type, and subclone among 124 antimicrobial-resistant** ***Escherichia coli*** **and** ***Klebsiella pneumoniae*** **isolates from long-term care facility residents**

**Organism**	**PFGE cluster**	**No. of isolates**	***bla*** **genes, no. of isolates**				**ST** ^**a**^ **and subclone (no. of isolates)**
			***bla***_**CTX-M-group-1**_^**b**^	***bla***_**TEM**_	***bla***_**SHV**_	***bla***_**OXA-1**_	
*E. coli*	EcA^c^	91	91	0	0	79	ST131 *H*30-Rx(91)
*E. coli*	EcA1^d^	2	1	0	0	0	ST131 *H*30-Rx (1)
*E. coli*	EcB^e^	6	6	0	0	6	ST131 *H*30-Rx (6)
*K. pneumoniae*	KpA^f^	6	6	0	6	6	ST1236/ST48 (6)^g^

^a^ST = sequence type (from multilocus sequence typing). Inferred from PCR-based assays for *E. coli* and by PFGE similarity to reference strains of known sequence type (ST) for *K. pneumoniae* strains.

^b^Although other *bla*_CTX-M_ genes were tested for including *bla*_CTX-M-group-2_, *bla*_CTX-M-group-8_, *bla*_CTX-M-group-9_, and *bla*_CTX-M-group-25_, none were detected other than *bla*_CTX-M-group-1_.

^c^EcA = ESBL-EC (n = 91) represented in PFGE Cluster A and similar to representative of UK Strain A and international PFGE812.

^d^EcA1 = ESBL-EC (n = 1) demonstrating 83% similarity to strains in PFGE Cluster A.

^e^EcB = ESBL-EC (n = 6) represented in PFGE Cluster B.

^f^KpA = ESBL-KP PFGE cluster similar to ST1236.

^g^ST1236 is a single-locus variant of ST48; both are part of clonal complex 43.

**Figure 1:**
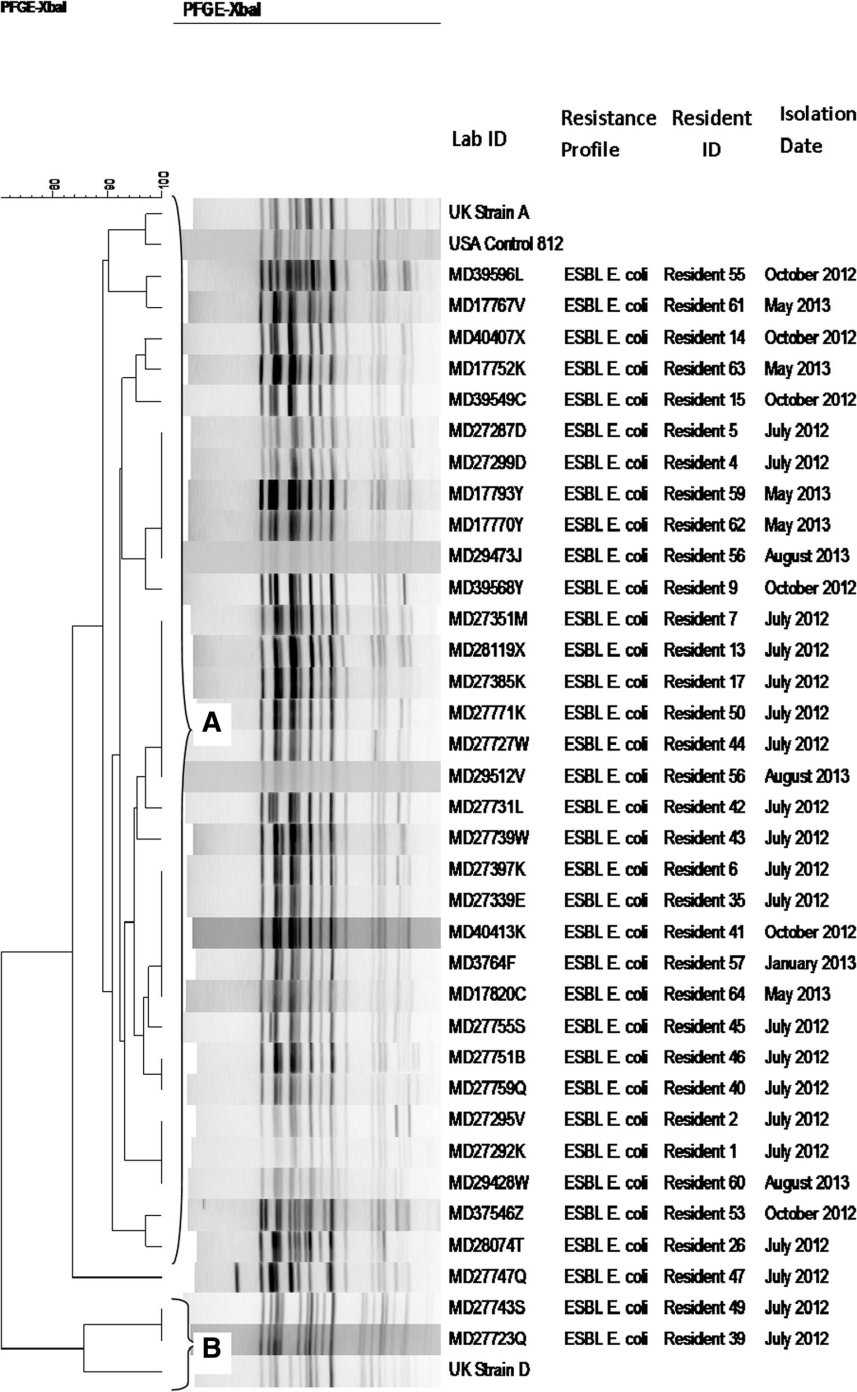
**Dendrogram of** ***Xba*** **I pulsed-field gel electrophoresis (PFGE) profiles for** ***Escherichia coli*** **isolates.** One representative of each PFGE type per subject was included in the dendrogram, which includes extended-spectrum β-lactamase-producing *E. coli* (ESBL-EC) (n = 35) isolates, plus reference strains representing UK Strains A and D, and international pulsotype labelled USA Control 812. Reference strains with ≥ 85% profile similarity to study isolates are shown. The dendrogram was generated using the unweighted pair group method with arithmetic mean (UPGMA) algorithm based on Dice similarity coefficients. Two PFGE clusters **(A and B)** were defined based on ≥ 85% profile similarity. Isolates labelled “ESBL E. coli” represent ESBL-EC.

In a PFGE dendrogram that included each colonised resident’s first ESBL-EC isolate (n = 35) and selected reference isolates, the study isolates clustered with the three UK and international reference ST131 strains at 72% profile similarity (Figure 1). The *H*30-Rx ST131 isolates were divided into two clusters (clusters A and B), each with ≥ 85% profile similarity. Cluster A comprised 91 isolates from 33 participants and included reference isolates for UK strain A and international PFGE812. Cluster B comprised six isolates from two participants and UK strain D.

### Detection and characterisation of ESBL- producing *K. pneumoniae*

**Figure 2:**
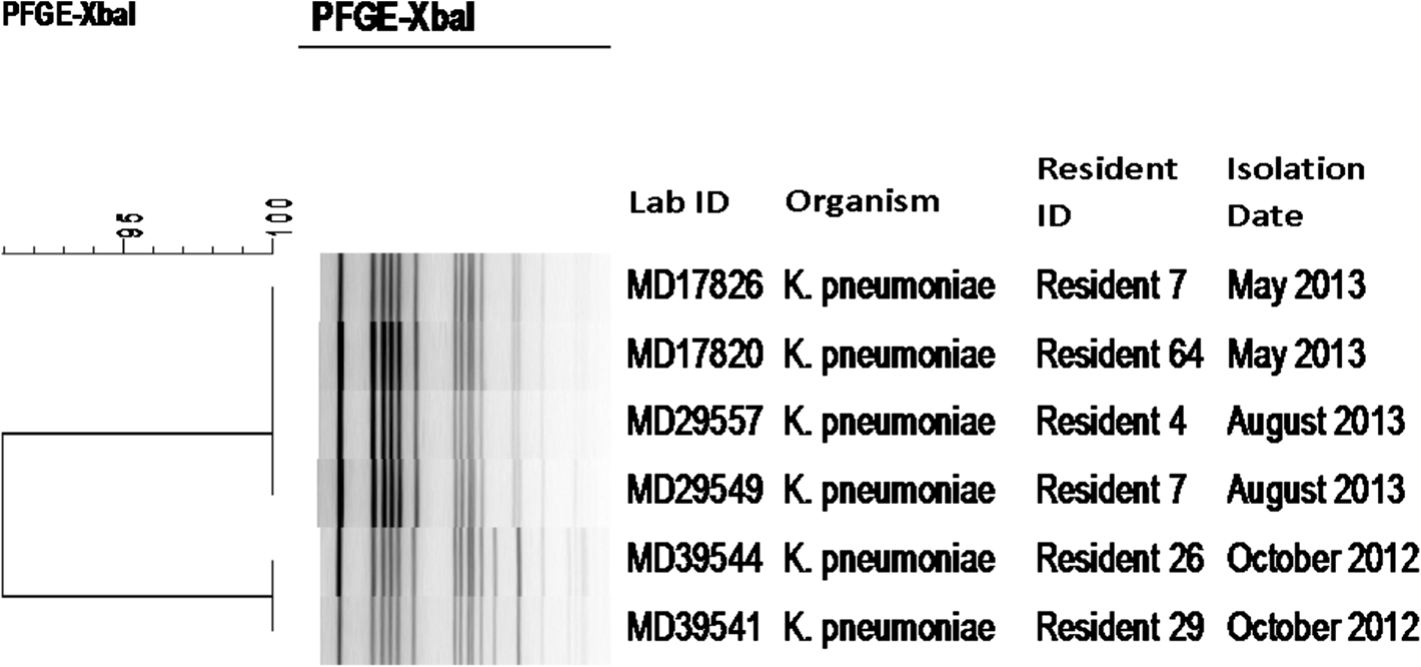
**Dendrogram of** ***Xba*** **I pulsed-field gel electrophoresis (PFGE) profiles for** ***Klebsiella pneumoniae*** **isolates.** PFGE dendrogram of the six ESBL-producing *Klebsiella pneumoniae* isolates, from 5 participants from October 2012 – August 2013. The dendrogram was generated using the unweighted pair group method with arithmetic mean (UPGMA) algorithm based on Dice similarity coefficients. All profiles are ≥ 91% similar.

At baseline, none of the participants tested positive for ESBL*-*KP; however, five subsequently tested positive*,* including three from a high dependency area and two from a low dependency area. The positive tests occurred at study months 3, 6, 9, and 12, in 2, 0, 2, and 2 participants, respectively (Table 2). Of the five ESBL*-*KP-colonised individuals, four were also colonised with ESBL-EC, therefore in total there were 36 residents colonised with ESBL-producing *Enterobacteriaceae*. None of the five ESBL*-*KP-colonised individuals had previously been identified as an ESBL carrier and only one had been hospitalised or received antimicrobials within three months of first being detected positive. That individual was newly admitted to the LTCF in May 2013 with ESBL-KP already present in the LTCF. All six ESBL-KP isolates were resistant to ciprofloxacin and gentamicin, and susceptible to piperacillin/tazobactam, cefoxitin, ertapenem, and meropenem. The six isolates were 91% similar by PFGE (Figure 2) and positive for *bla*_CTX-M group 1_, *bla*_SHV_, and *bla*_OXA-1_ (Table 3). The two isolates collected in October 2012 from different participants were indistinguishable by PFGE. The four subsequent isolates, collected from three participants in May and August 2013 (one patient was positive in both May and August), were indistinguishable from one another by PFGE but differed from the previous isolates by 3 bands (Figure 2). In comparison with a collection of ESBL-KP isolates from other regions of Ireland (unpublished data, D. Morris) the present isolates exhibited 86% PFGE profile similarity to reference isolates from *K. pneumoniae* clonal complex (CC) CC43 (i.e., ST48 and ST123)*.*

### Detection and characterisation of CPE, VRE, and MRSA

Carbapenemase-producing *Enterobacteriaceae* were not detected and vancomycin-resistant *E. faecium* were detected in only two participants. MRSA were detected in 17 (27%) of the 64 participants, including 11 (65%) by the first nasal swab, and six (35%) by subsequent nasal swabs. The 17 MRSA colonised residents comprised 13 baseline participants; 8 of 13 positive on the first round of screening, and four new participants (Table 2). Of the 17 (27% of 64) total participants colonised with MRSA at some point, six (35%) were positive for MRSA on two or more subsequent tests after their first positive test. Five (29%) of 17 MRSA-positive participants had been recognised as such prior to their first positive study test based on clinical samples from wound (n = 2) or nasal/body swabs (n = 3).

### Co-colonisation with multiple antimicrobial-resistant organisms

Co-colonisation with different AROs was common, with 14/36 (39%) ESBL-producing *Enterobacteriaceae*-positive participants also positive for MRSA, compared with 3/28 (11%) non-ESBL-colonised participants (P = 0.008). The two VRE-colonised participants were also colonised with ESBL-EC and MRSA. Of the 14 participants’ positive for both ESBL-producing *Enterobacteriaceae* and for MRSA, 10 were high dependency with a Barthel Index score of 25 or less indicating immobility and no level of ability/requiring major help to perform activities of daily living. The remaining four participants had Barthel Index Scores ranging from 30 – 100, with only 1 participant being able to perform all activity of daily living independence from any help.

### Environmental sampling

Four bedrooms and two common bathrooms were sampled based on the occupants’ colonisation status and care area; one with ESBL-EC, VRE and MRSA, one with ESBL-EC and ESBL-KP, one with ESBL-EC only, one with ESBL-KP only, and one occupant did not have detectable colonisation with any of the target organisms. MRSA was recovered from 12/69 (17%) environmental swabs, and from 5/7 (71%) rooms sampled. The only two rooms in which MRSA was not detected included one shared toilet facility and the bedroom of a resident in whom colonisation with ESBL-producing *Enterobacteriaceae*, VRE, and MRSA was not detected. Among the five MRSA-positive rooms, MRSA was recovered at least once from each of the 11 types of surfaces sampled, except for tap handles, railing beside the toilet, and the on-call button. ESBL-producing *Enterobacteriaceae* and VRE were not detected in environmental cultures.

### Longitudinal analysis of ESBL and MRSA colonisation

For ESBL-producing *Enterobacteriaceae*, receipt of any antimicrobial in the previous 3 months more than doubled the odds of colonisation (odds ratio 2.4 (1.5-3.8)). In a more detailed model that included antimicrobial class, use in the previous three months of broad-spectrum beta-lactam antimicrobials (OR 2.1 (1.2-3.4) or nitrofurantoin (OR 3.5 (1.1-11.3) was independently associated with ESBL colonisation. Care area was also an independent risk factor, with participants in (high-dependency) areas 1 and 2 having a nearly four-fold increased odds of ESBL colonisation (OR 3.9 (1.6-9.7).

For MRSA, receipt of any antimicrobials in the previous three months conferred a 3.1-fold (1.1-9.1) higher odds of testing MRSA-positive. Care area was also independently associated with MRSA colonisation, with participants in care areas 1 and 2 having a 6.9-fold (1.6-29.3) higher odds of testing MRSA-positive. Additional independent correlates of MRSA colonization included previous MRSA positivity (OR 17.7 (3.8-83.1)) and age (OR 1.12 (1.0-1.2)). No specific antimicrobial class was independently associated with MRSA colonisation.

## Discussion and conclusions

This is the first study to our knowledge that provides longitudinal data on colonisation with AROs and risk factors for colonisation in a LTCF. The need for such studies has been recognised as urgent in a recent editorial on *E. coli* ST131 [31]. Our study yielded nine key findings. First, ESBL-EC were detected in more than 50% of participants of the study LTCF. Second, MRSA were detected in more than 25% of participants, and co-colonisation of ESBL-EC and MRSA was common. Third, care area (corresponding with level of dependency) and antimicrobial use were risk factors for carriage of ESBL-EC and MRSA. Fourth, all ESBL-EC isolates belonged to the *H*30-Rx ST131 subclone. Fifth, PFGE divided the *H*30-Rx subclone into two clusters, one of which included UK Strain A and a representative of the international PFGE812 group, the other included UK strain D. Sixth, all ESBL-EC and ESBL-KP contained *bla*_CTX-M group 1_. Seventh, once subjects acquired ESBL-EC they tended to remain colonised with the same strain. Eight, we observed that although ESBL-producing *Enterobacteriaceae* were recovered from a much higher proportion of participants than MRSA, ESBL-producing *Enterobacteriaceae* was not detected in the environment, whereas detection of MRSA was common. Finally, 39% of residents did not become colonised with ESBL-EC or MRSA at any time, despite sharing the same LTCF with colonised residents from up to a year. These findings have significant implications for our understanding of the relationship between LTCF and the problem of acquired resistance to antimicrobial agents.

Several studies have assessed the prevalence of colonisation with ESBL-EC among LTCF participants. Our findings are broadly consistent with those of a previous study in Northern Ireland. However, our observed colonisation prevalence with ESBL-EC (56%) is higher than that study’s (41%) [20], and is much higher than noted in previous international LTCF surveillance studies, including studies from 2008–2010 and 2003–2012 from Sweden identified ESBL- EC in just 1/268 (0.004%) and 2/1131 (0.002%) residents respectively. Similarly, studies performed in 2010 and 2011 from Melbourne identified ESBL-EC in 14/119 (12%) and 12/115 (10%) of LTCF residents respectively [41-44].

MRSA colonisation was detected at some time in 27% of participants, which is similar to a previous study from Northern Ireland (23% of residents in 45 LTCFs), but higher than an earlier study performed in six LTCFs in Ireland between 1995 and 1996 (9% and 10% respectively) [23,45]. It is possible that 27% represents an underestimate of colonisation, since practical constraints limited our sampling to nasal swabs. A low prevalence of MRSA colonisation among LTCF residents was reported from Sweden, the Netherlands, and Denmark [46-48]. However, these countries have a low proportion of MRSA bloodstream infections compared with Ireland [17], which suggests that MRSA is less prevalent overall as a healthcare-associated organism in those countries compared with Ireland.

Co-colonisation with ESBL-producing *Enterobacteriaceae* and MRSA was common (39%). Although VRE colonisation was uncommon (3%), it likewise was associated with ESBL-EC and MRSA colonisation. Elsewhere, VRE colonisation of LTCF residents was not detected in Belgium, but has been reported in Australia (2%), Israel (10%), and in the USA (4 to 45%) [42,49-52]. Similarly, Donskey and colleagues (2003) found that, in a Department of Veterans Affairs medical centre (which includes an acute care facility and a separate nursing home), colonisation with MRSA and ceftazidime-resistant Gram-negative bacilli was more prevalent among VRE-positive than VRE-negative patients [53]. Thus our findings of co-colonisation are consistent with existing literature.

Colonisation with ESBL-producing *Enterobacteriaceae* and MRSA were associated with residence in particular care areas within the LTCF and with antimicrobial consumption. Specifically, participants in care areas 1 and 2 were 3.9 odds more likely to be ESBL colonised, and 6.9 odds more likely to be MRSA colonised. The association with care area may plausibly reflect the interaction of a number of factors associated with providing care for the most dependent residents, including greater resident vulnerability, and more frequent contact with healthcare workers. This reflects the challenges of containing spread of AROs while providing appropriate social, psychological, and physical care for highly dependent residents in a LTCF.

Participants also had a 2.4-fold higher odds of ESBL colonisation and 3.1-fold higher odds of MRSA colonisation if they received antimicrobial in the three months before sampling. Fifty-two percent of participants received antimicrobials during the one-year study period. Broad spectrum beta-lactam antimicrobials (included amoxicillin, co-amoxiclav, and cefuroxime) were the most frequently consumed antimicrobials, with amoxicillin-clavulanic acid accounting for the majority. This is consistent with the European Surveillance of Antimicrobial Consumption point prevalence studies, which identified beta-lactam antimicrobials and penicillins as the most common antimicrobials prescribed in Irish LTCFs [54]. The 2013 HALT survey for Ireland reported 9% and 11% respectively as the median overall antimicrobial use prevalence in general nursing homes (n = 103) and mixed care facilities (n = 26), both with length-of-stay > 12 months [55]. Likewise, in 722 European LTCFs the most commonly prescribed agents were amoxicillin-clavulanic acid (12.7%), nitrofurantoin (10.4%), trimethoprim (9.9%), amoxicillin (7.3%), and ciprofloxacin (6.9%) [24]. A higher point prevalence of antimicrobial prescribing (16%) has been reported in LTCFs in other European studies [56,57]. Benoit *et al.* [58] reported 42% of residents in 73 LTCFs as receiving antimicrobials in the previous six months in the USA.

Given that third-generation cephalosporins and fluoroquinolones were used infrequently in this facility, it appears that selective pressure from these agents is not likely to be a major factor in the high prevalence of colonisation with ESBL-producing *Enterobacteriaceae* or MRSA. However, we note that in this LTCF the dominant ESBL-*Enterobacteriaceae* and MRSA are resistant to broad-spectrum beta-lactams, the most commonly consumed antimicrobials in the LTCF; thus, the overall association of colonisation with antimicrobial consumption is not surprising from a microbiological perspective.

It was surprising that nitrofurantoin was independently associated with ESBL colonisation (OR 3.4 (1.1-11.3)), since it is generally considered to have limited selective impact on gut flora [59]. However, record review indicated that 8/9 participants who received nitrofurantoin prior to sampling had already been identified as ESBL-positive before the nitrofurantoin was prescribed, based on clinically-submitted urine samples. Since nitrofurantoin use in these patients was most likely guided by prior susceptibility test reports, and since nitrofurantoin is one of few oral antimicrobials active against ESBL-EC, this association can be explained on that basis.

The finding that all ESBL-EC isolates represented the ST131 subclone contributes to the evidence that dissemination of ST131 is a global public health problem in LTCFs. Banerjee *et al.* [60] reported that, among consecutive *E. coli* clinical isolates from Rochester, Minnesota, ST131 accounted for 80/299 (27%) of isolates from healthcare or community-associated infections, but for fully 28/37 (76%) of isolates from LTCFs. Rooney *et al.* and Dhanji *et al.* reported ST131 representing 99% of ESBL-EC collected from residents of LTCFs in Northern Ireland [20,61]. The proportion of ST131 we encountered among faecal ESBL-EC was similar to these reports, and higher than in other international studies of LTCF residents [20,60,61]. The predominance of ST131 in LTCFs suggests that such facilities may serve as reservoirs for dissemination of ST131. This problem is likely to be exacerbated by growing number of LTCF residents, which currently is approximately 19,800 (2012) in Ireland, 3.7 million in the EU (2010), and 1.5 million in the United States (2009) [24,25,31].

Genomic diversity within ST131 has been described previously based on sequencing the *fimH*, *gyrA,* and *parC* genes, PFGE, and whole genome analysis [3,6,7]. The *H*30 subclone and its *H*30-Rx subset have been identified as the major lineages of *E. coli* associated with antimicrobial-resistant infections. Colpan *et al.* [38] found that the *H*30 subclone as a whole accounted for ≥ 95% of fluoroquinolone-resistant and ESBL-producing ST131 isolates from US veterans. Price *et al.* [7] and Banerjee *et al*. [5] reported a high proportion of ESBL-EC as belonging to the *H30*-Rx subset; 91% and 92% respectively [5,7]. Similarly, in the present study all ESBL-EC belonged to the *H*30-Rx subclone. The *H*30-Rx subclone has a reported association with sepsis, suggesting that virulence may be contributing to its epidemiologic success [5,7].

Johnson *et al.* [3] and Colpan *et al.* [38] identified various pulsotypes within ST131, with PFGE968, PFGE800, and PFGE812 being the most prevalent [3,38]. Here, 91/98 (93%) of the *H*30-Rx isolates formed a single PFGE cluster, at the ≥ 85% similarity level. This group includes the ST131 UK epidemic strain A and a representative of international PFGE812 (Figure 1). The latter two reference strains represent, respectively, the most prevalent ST131 variant in the UK [40,62] and the most prevalent European-associated ST131 pulsotype in a large private PFGE library (unpublished data, J. R. Johnson) [3]. Thus, the predominance of this clonal group within the study LTCF is part of the international dissemination of the *H*30Rx ST131 subclone. However, six of the remaining eight *H*30-Rx isolates, from two participants, formed a distinct PFGE cluster, Cluster B (Figure 1). It is of interest that this variant was not entirely displaced by the predominant variant; thus, two variants of the *H*30-Rx subclone were circulating in the LTCF.

Our finding of high prevalence of *bla*_CTX-M_ group 1 is consistent with a previous reports from Banjeree *et al.* [5] and Price *et al.* [7] demonstrating a high prevalence (91%) of *bla*_CTX-M-15_ within the *H*30-Rx subclone [5,7]. Additionally, we found that most isolates (87%) also carried *bla*_OXA-1,_ which was associated with co-amoxiclav resistance, reflecting inefficient inhibition of OXA enzymes by clavulanic acid [63]. This finding may be relevant to the success of this clonal group given the frequent consumption of co-amoxiclav.

Although few, the ESBL-KP in our study were 91% similar by PFGE (Figure 2). All carried *bla*_CTX-M group 1_ and *bla*_OXA-1_ and were resistant to co-amoxiclav. The six ESBL-KP strains were 86% similar to other Irish isolates belonging to *K. pneumoniae* clonal CC43 (i.e., ST48 and ST123) This is consistent with clonal dissemination of ESBL-KP in Ireland, similar to that described elsewhere [64]. Various clonal groups of *K. pneumoniae* have been reported worldwide, such as CC43, which is commonly associated with resistance enzymes such as TEM-3, SHV-12, and CTX-M-15 [10,12]. We detected ESBL*-*KP for the first time in this LTCF three months after commencing the study. Given the propensity for dissemination of such strains of *K. pneumoniae* [65], it seems surprising that after nine months of follow-up only five participants had evidence of colonisation.

The duration of persistence of colonisation with ESBL-producing *Enterobacteriaceae* has previously been reported. The median time to clearance of ESBL-producing *Enterobacteriaceae* after hospital discharge was 6.6 months in a French study [66] and 98 days (i.e., approximately 3 months) in a study from Thailand [67]. A Swedish study by Titelman *et al*. [68] concluded that clearance of ESBL-producing *Enterobacteriaceae* has not occurred in many patients (43%) at 12 months after infection and that persistence is associated with phylogroup B2 *E. coli* carrying *bla*_CTX-M group 9_. This study also demonstrated that failure to detect the organism on sampling does not reliably establish clearance [68]. However, these studies did not address residents of LTCFs.

We found that spontaneous clearance of colonisation of *E. coli* carrying *bla*_CTX-M group 1_ was uncommon; 75% of ESBL-EC colonised participants who had two or more subsequent samples after their initial positive swab remained positive. Moreover, despite the presence within this LTCF of two different PFGE variants of ESBL-EC ST131, the variant identified in a given resident was stable over time. Thus, participants tended to remain colonised with the same ESBL-EC strain for an extended period. Furthermore, two initially ESBL-EC-colonised participants in whom ESBL-EC ST131 was not detected at 3 or 6 months reverted to being positive at 9 and 12 months, in both cases with isolates that were indistinguishable from or ≥ 95% similar to the previous isolates by PFGE. This is consistent with persistence, or auto-reinfection. As no environmental contamination with ESBL-producing *Enterobacteriaceae* was detected, auto-reinfection does not appear to be from an environmental reservoir but more likely from resident-resident interactions or resident-health care worker interactions. This may also indicate the need for caution in accepting failure to detect colonisation as confirmation of clearance.

Persistent colonisation was also common for MRSA. Of the 17 MRSA-colonised participants 73% of those who had two or more subsequent samples remained positive. Consistent with standard practice in LTCF in Ireland this LTCF had no programme of routine resident MRSA decolonisation.

Regarding environmental contamination, MRSA was recovered much more commonly from the environment than was ESBL-EC, despite being recovered from a smaller proportion of participants, which is consistent with previous findings [69]. The greater frequency of environmental detection of MRSA compared with ESBL-*E. coli* may reflect more intense shedding of MRSA or better environmental survival. The environmental detection of MRSA compared with no ESBL-EC environmental contamination may reflect more intense shedding of MRSA or better environmental survival.

Interestingly, a large number of residents did not become colonised with ESBL-EC or MRSA throughout the year. This occurred despite an absence of restrictions on social interactions for residents colonised with MRSA or ESBL-*Enterobacteriaceae*, and frequent environmental contamination with MRSA. It is clear that, as the inverse of risk factors for colonisation, those who do not become colonised were less likely to have consumed antimicrobial agents and to reside in the high-dependency areas. However, other factors may contribute to colonisation resistance. This phenomenon clearly merits further study.

The study has several limitations. First, although initial study enrolment was high and retention was good, both were incomplete, with represents one of many challenges in conducting a longitudinal LTCF-based study. Second, antimicrobial exposure may have been underestimated, since antimicrobial use during hospital admission was not captured. Third, although the observed clonality of the ESBL*-*EC and ESBL-KP study isolates supports dissemination within this LTCF, it is not possible from the current analysis to differentiate definitively between acquisition of ESBL-producing organisms within the LTCF vs. during episodes of hospitalisation or via other exposures. In future studies, emerging higher-resolution typing methods such as whole-genome sequencing could be used to better clarify transmission pathways [70].

Despite those limitations this study represents a significant contribution to research in this area given the lack of longitudinal studies in LTCFs and there are several novel and important findings. Antimicrobial prescribing in LTCFs is an immediately modifiable risk factor for colonisation with AROs. We demonstrate the clonality and temporal stability of ESBL-producing *Enterobacteriaceae* within a single LTCF. Although multiple ST131 variants were detected, all belong to the *H*30*-*Rx subclone [5,7]. Colonised participants tended to retain the same pulsotype over an extended period. We report the first identified ESBL-KP in an Irish LTCF. We note that although present in 5 participants this organism has not disseminated to the same extent as ESBL-EC (n = 35). Further studies are needed to clarify the acquisition, retention, and dissemination of antimicrobial-resistant clones within LTCFs and to establish if there are other characteristics of such individuals, e.g., the gut microbiota, that may contribute to such colonisation resistance.

## References

[1] Spellberg B, Guidos R, Gilbert D, Bradley J, Boucher HW, Scheld WM (2008). The epidemic of antibiotic-resistant infections: a call to action for the medical community from the Infectious Diseases Society of America. Clin Infect Dis.

[2] Irish EARS-Net Steering Group (2014). EARS-Net report, quarter 4 2013.

[3] Johnson JR, Nicolas-Chanoine M-H, DebRoy C, Castanheira M, Robiscek A, Hansen G (2012). Comparison of *Escherichia coli* ST131 pulsotypes, by epidemiologic traits, 1967–2009. Emerg Infect Dis.

[4] Nicolas-Chanoine M-H, Blanco J, Leflon-Guibout V, Demarty R, Alonso MP, Caniça MM (2008). Intercontinental emergence of *Escherichia coli* clone O25:H4-ST131 producing CTX-M-15. J Antimicrob Chemother.

[5] Banerjee R, Robicsek A, Kuskowski MA, Porter S, Johnston BD, Sokurenko E (2013). Molecular epidemiology of *Escherichia coli* sequence type 131 and its H30 and H30-Rx subclones among extended-spectrum-β-lactamase-positive and -negative *E. coli* clinical isolates from the Chicago region, 2007 to 2010. Antimicrob Agents Chemother.

[6] Johnson JR, Tchesnokova V, Johnston B, Clabots C, Roberts PL, Billig M (2013). Abrupt emergence of a single dominant multidrug-resistant strain of *Escherichia coli*. J Infect Dis.

[7] Price LB, Johnson JR, Aziz M, Clabots C, Johnston B, Tchesnokova V (2013). The epidemic of extended-spectrum-β-lactamase-producing *Escherichia coli* ST131 is driven by a single highly pathogenic subclone, H30-Rx. mBio.

[8] Qi Y, Wei Z, Ji S, Du X, Shen P, Yu Y (2011). ST11, the dominant clone of KPC-producing *Klebsiella pneumoniae* in China. J Antimicrob Chemother.

[9] Nielsen JB, Skov MN, Jørgensen RL, Heltberg O, Hansen DS, Schønning K (2011). Identification of CTX-M15-, SHV-28-producing *Klebsiella pneumoniae* ST15 as an epidemic clone in the Copenhagen area using a semi-automated Rep-PCR typing assay. Eur J Clin Microbiol Infect Dis.

[10] Marcade G, Brisse S, Bialek S, Marcon E, Leflon-Guibout V, Passet V (2013). The emergence of multidrug-resistant *Klebsiella pneumoniae* of international clones ST13, ST16, ST35, ST48 and ST101 in a teaching hospital in the Paris region. Epidemiol Infect.

[11] Shin J, Soo Ko K (2013). Single origin of three plasmids bearing *blaCTX-M-15* from different *Klebsiella pneumoniae* clones. J Antimicrob Chemother.

[12] Wang G, Huang T, Surendraiah PKM, Wang K, Komal R, Zhuge J (2013). CTX-M β-lactamase-producing *Klebsiella pneumoniae* in suburban New York City, New York, USA. Emerg Infect Dis.

[13] Morris D, McGarry E, Cotter M, Passet V, Lynch M, Ludden C (2012). Detection of OXA-48 carbapenemase in the pandemic clone *Escherichia coli* O25b:H4-ST131 in the course of investigation of an outbreak of OXA-48-producing *Klebsiella pneumoniae*. Antimicrob Agents Chemother.

[14] Steinmann J, Kaase M, Gatermann S, Popp W, Steinmann E, Damman M (2011). Outbreak due to a *Klebsiella pneumoniae* strain harbouring KPC-2 and VIM-1 in a German university hospital, July 2010 to January 2011. Euro Surveill.

[15] Roche C, Cotter M, O’Connell N, Crowley B (2009). First identification of class A carbapenemase-producing *Klebsiella pneumoniae* in the Republic of Ireland. Euro Surveill.

[16] Morris D, Boyle F, Ludden C, Condon I, Hale J, O’Connell N (2011). Production of KPC-2 carbapenemase by an *Escherichia coli* clinical isolate belonging to the international ST131 clone. Antimicrob Agents Chemother.

[17] European Antimicrobial Resistance Surveillance Network (EARS-Net) database. Stockholm: European Centre for Disease Prevention and Control. Available from: http://www.ecdc.europa.eu/en/healthtopics/antimicrobial_resistance/database/Pages/table_reports.aspx

[18] Monecke S, Coombs G, Shore AC, Coleman DC, Akpaka P, Borg M (2011). A field guide to pandemic, epidemic and sporadic clones of methicillin-resistant *Staphylococcus aureus*. PLoS One.

[19] Pelly H, Morris D, O’Connell E, Hanahoe B, Chambers C, Biernacka K (2006). Outbreak of extended spectrum beta-lactamase producing *E. coli* in a nursing home in Ireland, May 2006. Euro Surveill.

[20] Rooney PJ, O’Leary MC, Loughrey AC, McCalmont M, Smyth B, Donaghy P (2009). Nursing homes as a reservoir of extended-spectrum β-lactamase (ESBL)-producing ciprofloxacin-resistant *Escherichia coli*. J Antimicrob Chemother.

[21] Burke L, Humphreys H, Fitzgerald-Hughes D (2012). The revolving door between hospital and community: extended-spectrum beta-lactamase-producing *Escherichia coli* in Dublin. J Hosp Infect.

[22] Brabazon E, Carton M, Dornikova G, Bedford D (2012). Epidemiology and resistance patterns in urinary pathogens from long-term care facilities and GP populations. Ir Med J.

[23] Baldwin NS, Gilpin DF, Hughes CM, Kearney MP, Gardiner DA, Cardwell C (2009). Prevalence of methicillin-resistant *Staphylococcus aureus* colonization in residents and staff in nursing homes in Northern Ireland. J Am Geriatr Soc.

[24] Suetens C (2012). Healthcare-associated infections in European long-term care facilities: how big is the challenge?. Euro Surveill.

[25] Information Unit, Department of Health Ireland (2014). Long-stay activity statistics 2012.

[26] Fennell J, Vellinga A, Hanahoe B, Morris D, Boyle F, Higgins F (2012). Increasing prevalence of ESBL production among Irish clinical *Enterobacteriaceae* from 2004 to 2008: an observational study. BMC Infect Dis.

[27] Bailey AM, Weant KA, Baker SN (2013). Prevalence and risk factor analysis of resistant *Escherichia coli* urinary tract infections in the emergency department. Pharm Pract (Granada).

[28] Friedmann R, Raveh D, Zartzer E, Rudensky B, Broide E, Attias D (2009). Prospective evaluation of colonization with extended-spectrum β-lactamase (ESBL)–producing *Enterobacteriaceae* among patients at hospital admission and of subsequent colonization with ESBL-producing *Enterobacteriaceae* among patients during hospitalization. Infect Control Hosp Epidemiol.

[29] Catry B, Latour K, Jans B, Vandendriessche S, Preal R, Mertens K (2014). Risk factors for methicillin resistant *Staphylococcus aureus*: a multi-laboratory study. PLoS One.

[30] Karki S, Houston L, Land G, Bass P, Kehoe R, Borrell S (2012). Prevalence and risk factors for VRE colonisation in a tertiary hospital in Melbourne, Australia: a cross sectional study. Antimicrobial ResistInfect Contr.

[31] Lautenbach E (2013). Editorial commentary: flying under the radar: the stealth pandemic of *Escherichia coli* sequence type 131. Clin Infect Dis.

[32] Centers for Disease Control and Prevention (2009). Laboratory protocol for detection of carbapenem-resistant or carbapenemase-producing *Klebsiella spp*. and *E. coli* from rectal swabs.

[33] Health Protection Surveillance Centre (2011). National pilot study of carbapenemase-producing carbapenem resistant *Enterobacteriaceae* (CRE) in critical care units in the Republic of Ireland. Study protocol. Version 1.4.

[34] EUCAST: European Committee on Antimicrobial Susceptibility Testing (2013). Breakpoint tables for interpretation of MICs and zone diameters. Version 3.1. February 2013.

[35] Woodford N, Fagan EJ, Ellington MJ (2006). Multiplex PCR for rapid detection of genes encoding CTX-M extended-spectrum β-lactamases. J Antimicrob Chemother.

[36] Dallenne C, Da Costa A, Decré D, Favier C, Arlet G (2010). Development of a set of multiplex PCR assays for the detection of genes encoding important β-lactamases in *Enterobacteriaceae*. J Antimicrob Chemother.

[37] Clermont O, Dhanji H, Upton M, Gibreel T, Fox A, Boyd D (2009). Rapid detection of the O25b-ST131 clone of *Escherichia coli* encompassing the CTX-M-15-producing strains. J Antimicrob Chemother.

[38] Colpan A, Johnston B, Porter S, Clabots C, Anway R, Thao L (2013). *Escherichia coli* sequence type 131 (ST131) sub-clone H30 as an emergent multidrug-resistant pathogen among US veterans. Clin Infect Dis.

[39] Swaminathan B, Barrett TJ, Hunter SB, Tauxe RV (2001). PulseNet: the molecular subtyping network for foodborne bacterial disease surveillance, United States. Emerg Infect Dis.

[40] Woodford N, Carattoli A, Karisik E, Underwood A, Ellington MJ, Livermore DM (2009). Complete nucleotide sequences of plasmids pEK204, pEK499, and pEK516, encoding CTX-M enzymes in three major *Escherichia coli* lineages from the United Kingdom, all belonging to the international O25:H4-ST131 clone. Antimicrob Agents Chemother.

[41] Olofsson M, Toepfer M, Ostgren CJ, Midlov P, Matussek A, Lindgren PE (2013). Low level of antimicrobial resistance in *Escherichia coli* among Swedish nursing home residents. Scand J Infect Dis.

[42] Stuart RL, Kotsanas D, Webb B, Vandergraaf S, Gillespie EE, Hogg GG (2011). Prevalence of antimicrobial-resistant organisms in residential aged care facilities. Med J Aust.

[43] Sundvall P-D, Elm M, Gunnarsson R, Mölstad S, Rodhe N, Jonsson L (2014). Antimicrobial resistance in urinary pathogens among Swedish nursing home residents remains low: a cross-sectional study comparing antimicrobial resistance from 2003 to 2012. BMC Geriatr.

[44] Lim CJ, Cheng AC, Kennon J, Spelman D, Hale D, Melican G (2014). Prevalence of multidrug-resistant organisms and risk factors for carriage in long-term care facilities: a nested case–control study. J Antimicrob Chemother.

[45] O’Sullivan NP, Keane CT (2000). The prevalence of methicillin-resistant *Staphylococcus aureus* among the residents of six nursing homes for the elderly. J Hosp Infect.

[46] Andersson H, Lindholm C, Iversen A, Giske CG, Örtqvist Å, Kalin M (2012). Prevalence of antibiotic-resistant bacteria in residents of nursing homes in a Swedish municipality: healthcare staff knowledge of and adherence to principles of basic infection prevention. Scand J Infect Dis.

[47] Hoogendoorn M, Smalbrugge M, Stobberingh EE, van Rossum SV, Vlaminckx BJ, Thijsen SF (2013). Prevalence of antibiotic resistance of the commensal flora in Dutch nursing homes. J Am Med Dir Assoc.

[48] Greenland K, Rijnders MIA, Mulders M, Haenen A, Spalburg E, Van De Kassteele J (2011). Low prevalence of methicillin-resistant *Staphylococcus aureus* in Dutch nursing homes. J Am Geriatr Soc.

[49] Jans B, Schoevaerdts D, Huang TD, Berhin C, Latour K, Bogaerts P (2013). Epidemiology of multidrug-resistant microorganisms among nursing home residents in Belgium. PLoS One.

[50] Benenson S, Cohen MJ, Block C, Stern S, Weiss Y, Moses AE (2009). Vancomycin-resistant enterococci in long-term care facilities. Infect Control Hosp Epidemiol.

[51] Elizaga ML, Weinstein RA, Hayden MK (2002). Patients in long-term care facilities: a reservoir for vancomycin-resistant enterococci. Clin Infect Dis.

[52] Pop-Vicas A, Mitchell SL, Kandel R, Schreiber R, D’Agata EM (2008). Multidrug-resistant Gram-negative bacteria in a long-term care facility: prevalence and risk factors. J Am Geriatr Soc.

[53] Donskey CJ, Ray AJ, Hoyen CK, Fuldauer PD, Aron DC, Salvator A (2003). Colonization and infection with multiple nosocomial pathogens among patients colonized with vancomycin-resistant *Enterococcus*. Infect Control Hosp Epidemiol.

[54] McClean P, Hughes C, Tunney M, Goossens H, Jans B (2011). Antimicrobial prescribing in European nursing homes. J Antimicrob Chemother.

[55] Roche F, Donlon S, Burns K (2014). Health Protection Surveillance Centre, point prevalence survey of healthcare-associated infections and antimicrobial use in long-term care facilities: May 2013 - Republic of Ireland national report: March 2014.

[56] Rummukainen M-L, Mäkelä M, Noro A, Finne-Soveri H, Lyytikäinen O (2013). Assessing prevalence of antimicrobial use and infections using the minimal data set in Finnish long-term care facilities. Am J Infect Control.

[57] McClean P, Tunney M, Gilpin D, Parsons C, Hughes C (2011). Antimicrobial prescribing in nursing homes in Northern Ireland: results of two point-prevalence surveys. Drugs Aging.

[58] Benoit SR, Nsa W, Richards CL, Bratzler DW, Shefer AM, Steele LM (2008). Factors associated with antimicrobial use in nursing homes: a multilevel model. J Am Geriatr Soc.

[59] McKinnell JA, Stollenwerk NS, Jung CW, Miller LG (2011). Nitrofurantoin compares favorably to recommended agents as empirical treatment of uncomplicated urinary tract infections in a decision and cost analysis. Mayo Clin Proc.

[60] Banerjee R, Johnston B, Lohse C, Porter SB, Clabots C, Johnson JR (2013). *Escherichia coli* sequence type 131 is a dominant, antimicrobial-resistant clonal group associated with healthcare and elderly hosts. Infect Control Hosp Epidemiol.

[61] Dhanji H, Doumith M, Rooney PJ, O’Leary MC, Loughrey AC, Hope R (2011). Molecular epidemiology of fluoroquinolone-resistant ST131 *Escherichia coli* producing CTX-M extended-spectrum β-lactamases in nursing homes in Belfast, UK. J Antimicrob Chemother.

[62] Lau SH, Kaufmann ME, Livermore DM, Woodford N, Willshaw GA, Cheasty T (2008). UK epidemic *Escherichia coli* strains A–E, with CTX-M-15 β-lactamase, all belong to the international O25:H4-ST131 clone. J Antimicrob Chemother.

[63] Paterson DL, Bonomo RA (2005). Extended-spectrum β-lactamases: a clinical update. Clin Microbiol Rev.

[64] Breurec S, Guessennd N, Timinouni M, Le TAH, Cao V, Ngandjio A (2013). *Klebsiella pneumoniae* resistant to third-generation cephalosporins in five African and two Vietnamese major towns: multiclonal population structure with two major international clonal groups, CG15 and CG258. Clin Microbiol Infect.

[65] Hilty M, Betsch BY, Bögli-Stuber K, Heiniger N, Stadler M, Küffer M (2012). Transmission dynamics of extended-spectrum β-lactamase–producing *Enterobacteriaceae* in the tertiary care hospital and the household setting. Clin Infect Dis.

[66] Birgand G, Armand-Lefevre L, Lolom I, Ruppe E, Andremont A, Lucet J-C (2013). Duration of colonization by extended-spectrum β-lactamase-producing *Enterobacteriaceae* after hospital discharge. Am J Infect Control.

[67] Apisarnthanarak A, Bailey TC, Fraser VJ (2008). Duration of stool colonization in patients infected with extended-spectrum beta-lactamase–producing *Escherichia coli* and *Klebsiella pneumoniae*. Clin Infect Dis.

[68] Titelman E, Hasan CM, Iversen A, Nauclér P, Kais M, Kalin M (2014). Fecal carriage of extended-spectrum β-lactamase-producing *Enterobacteriaceae* is common twelve months after infection and is related to strain factors. Clin Microbiol Infect.

[69] Ludden C, Cormican M, Austin B, Morris D (2013). Rapid environmental contamination of a new nursing home with antimicrobial-resistant organisms preceding occupation by residents. J Hosp Infect.

[70] Snitkin ES, Zelazny AM, Thomas PJ, Stock F, Program NCS, Henderson DK (2012). Tracking a hospital outbreak of carbapenem-resistant *Klebsiella pneumoniae* with whole-genome sequencing. Sci Transl Med.

